# Nanozymes for extracellular matrix and cell sheet engineering: biomedical potential and complementary enzymatic strategies

**DOI:** 10.3389/fmolb.2026.1900627

**Published:** 2026-08-20

**Authors:** Soon Mo Choi, Sum Mi Zo, Ankur Sood, Sung Soo Han

**Affiliations:** 1 Research Institute of Cell Culture, Yeungnam University, Gyeongsan, Republic of Korea; 2 School of Chemical Engineering, Yeungnam University, Gyeongsan, Republic of Korea

**Keywords:** cell sheet engineering, ECM crosslinking, immobilized enzymes, nanozymes, redox homeostasis, scaffold-free tissue engineering, tissue engineering

## Abstract

Cell sheet engineering has emerged as a scaffold-free biofabrication strategy that preserves intrinsic cell–cell and cell–extracellular matrix interactions, demonstrating significant translational potential in regenerative medicine, including cardiac patches, cartilage and skin reconstruction, and corneal transplantation. Despite these advances, thick or multilayered cell sheets remain constrained by diffusion related hypoxia, accumulation of reactive oxygen species, ECM destabilization, and insufficient mechanical reinforcement, all of which interact to compromise long term structural integrity. Rather than providing a conventional survey of recent developments, this conceptual integration review reframes these limitations as a coupled reaction–diffusion–mechanics problem. We examine how redox imbalance and structural insufficiency form a self-reinforcing failure network in thick scaffold-free constructs and propose a reciprocal dual-axis catalytic framework to address this instability. Nanozymes, through catalase-, peroxidase-, and superoxide dismutase-like catalytic activities, regulate oxidative stress, alleviate hypoxic amplification, and establish a permissive redox microenvironment that sustains cell viability and ECM synthesis. In parallel, immobilized enzyme systems facilitate collagen crosslinking and protease modulation, thereby reinforcing ECM architecture and enhancing tensile and shear resilience at the network level. Although nanozyme-based redox regulation and enzyme-mediated structural reinforcement have been extensively explored in oncology, antioxidant therapeutics, and industrial catalysis, their coordinated implications for scaffold-free cell sheet engineering have not been systematically integrated. By articulating a reciprocal dual-axis framework linking redox stabilization with biomechanical maturation, this review provides a systems-level design logic for improving thick cell sheet stability. Finally, we extend this catalytic paradigm beyond regenerative medicine to high-density sheet-like biofabrication systems, including cultured meat and cultured leather, positioning the framework as a transferable engineering principle across emerging biofabrication industries.

## 1 Introduction: nanozymes, immobilized enzymes, and scaffold-free tissue engineering

Cell sheet engineering represents a scaffold-free biofabrication strategy that enables the formation of functional tissues while preserving intrinsic cell to cell interactions and the endogenously produced extracellular matrix, referred to hereafter as ECM. Since the establishment of temperature responsive culture surfaces that allow non destructive recovery of intact cell sheets (Nagase et al., 2025; Duman et al., 2024), this approach has demonstrated substantial translational potential across diverse areas of regenerative medicine, including cardiac patches, cartilage and skin reconstruction, and corneal transplantation (Homma et al., 2020; Mauroux et al., 2024; Kobayashi et al., 2013). Unlike conventional porous scaffold-based tissue engineering, which relies on artificial matrices for structural support, cell sheet constructs derive their architectural integrity directly from self-assembled ECM and intercellular junctions. This intrinsic structural organization confers important advantages, including reduced immunogenicity and preservation of physiological cell to matrix interactions (Glazieva et al., 2023; Hu S. et al., 2023).

However, the structural autonomy that characterizes scaffold-free systems also entails inherent fragility. In the absence of an external framework, tissue stability depends entirely on the integrity of endogenous ECM and the metabolic state of resident cells. As tissue thickness increases through stacking or multilayer cultivation, oxygen transport becomes constrained by physical diffusion limits, increasing the likelihood of localized hypoxic regions within the interior. Diffusion restriction alters mitochondrial function and promotes accumulation of reactive oxygen species, ROS (Hamanaka and Chandel, 2009). Elevated ROS levels promote collagen oxidation and dysregulation of matrix metalloproteinases, MMPs (Yao et al., 2020), thereby disrupting the balance between ECM synthesis and degradation. Over time, these molecular perturbations reduce crosslink density within the ECM network and weaken mechanical strength, compromising the structural stability of scaffold-free tissues. Functional persistence of these constructs is therefore linked not only to cellular viability but also to the mechanical integrity and maturation state of the ECM architecture. To address these limitations, various engineering strategies have been explored, including oxygen releasing biomaterials (Camci-Unal et al., 2013), vascularization approaches (Janson et al., 2025), incorporation of external crosslinking agents (Chang et al., 2025), and growth factor mediated modulation of the cellular microenvironment (Ren et al., 2020). While these approaches enhance tissue viability or mechanical reinforcement, most have been developed within scaffold-based paradigms. Their direct translation to purely scaffold-free systems remains limited by fundamental differences in structural organization and load distribution mechanisms. Stabilizing scaffold-free tissues therefore requires regulatory strategies that operate intrinsically within the construct itself. Interventions must act locally within the tissue microenvironment without reliance on external load bearing materials. Within this framework, scaffold-free tissues are best understood as dynamic systems governed by the coupled interaction between redox homeostasis and matrix mechanics. Accumulation of ROS directly modifies ECM structure through oxidative damage to structural proteins, while variations in crosslink density and matrix stiffness reshape mechanotransduction pathways and cellular metabolic regulation. These processes are not independent but mutually reinforcing, forming a coupled biochemical and biomechanical feedback architecture. Accordingly, sustaining structural integrity in thick tissue assemblies requires an integrated catalytic strategy that simultaneously stabilizes the biochemical microenvironment and promotes biomechanical maturation.

In recent years, nanozymes have been extensively investigated across diverse pathological models for their capacity to regulate ROS and, in certain systems, to catalytically generate oxygen under hypoxic conditions (Zhou et al., 2025; Hu D. et al., 2023). In parallel, immobilized enzyme strategies have evolved as effective approaches for enhancing ECM crosslinking and reinforcing structural integrity through localized catalytic reactions (La et al., 2021; Chen J. et al., 2025). Despite their independent development in oxidative stress modulation and biomaterial mechanics, a systematic integration of these two catalytic dimensions within the structural context of scaffold-free cell sheet engineering remains limited.

**FIGURE 1:**
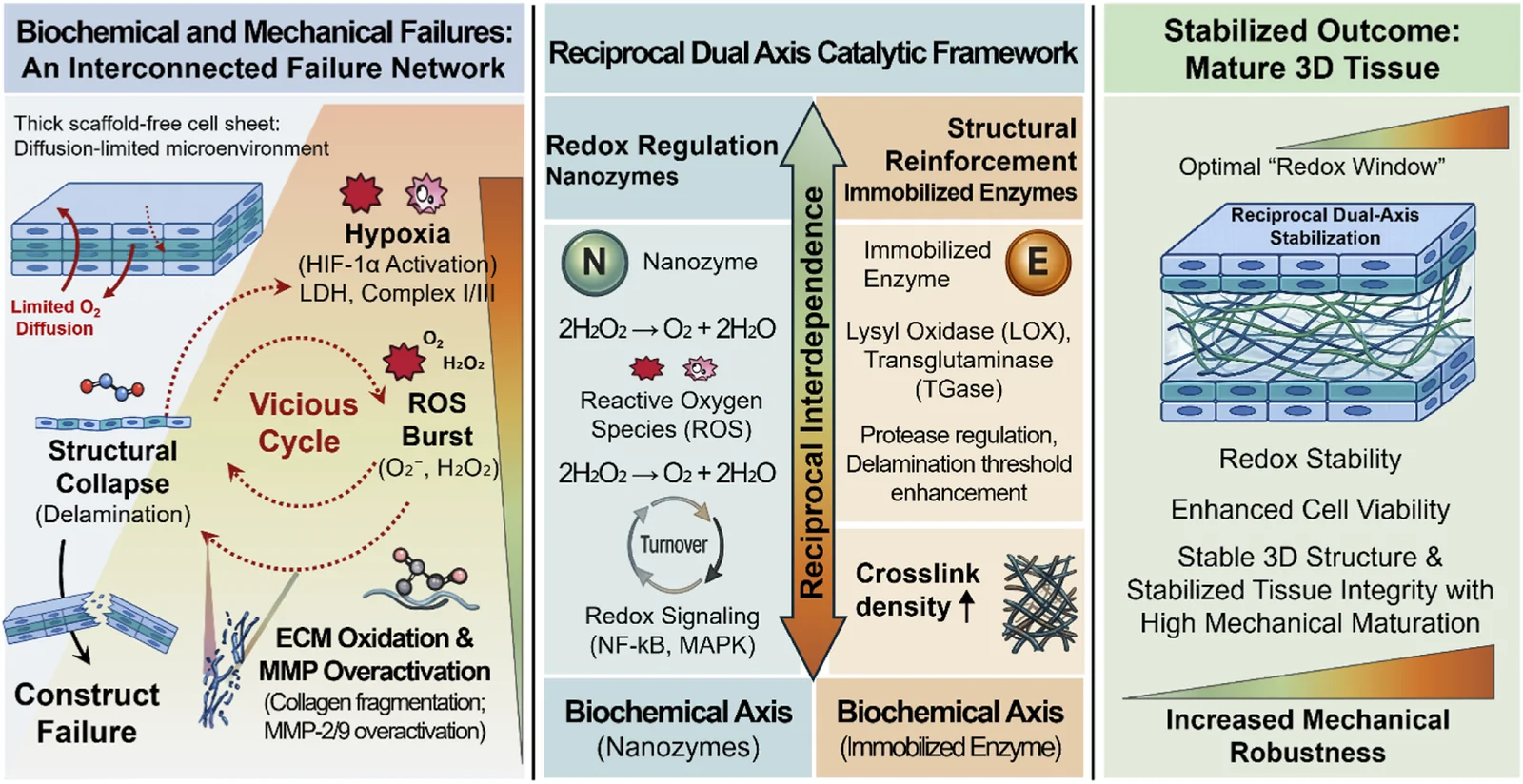
Conceptual overview of the reaction diffusion mechanics failure network and reciprocal dual axis catalytic stabilization strategy in scaffold-free cell sheets.

In this review, we reorganize the molecular failure mechanisms associated with cell sheet stacking through two interdependent dimensions, redox regulation and ECM mechanics. We introduce a Reciprocal Dual Axis Catalytic Framework for stabilizing scaffold-free tissues, as illustrated in Figure 1. Rather than treating hypoxia, ROS accumulation, ECM oxidation, and mechanical weakening as isolated pathological events, the framework conceptualizes them as components of a coupled biochemical and mechanical failure network that emerges under diffusion constrained conditions in thick constructs. This systems level perspective shifts the focus from correction of single pathways to regulation of network level vulnerability. The central component of Figure 1 delineates two concurrent and interdependent catalytic control axes. The redox regulation axis, mediated by nanozymes, restores biochemical permissiveness by limiting oxidative amplification. In parallel, the structural reinforcement axis, enabled by immobilized enzymes such as lysyl oxidase and transglutaminase, promotes crosslink formation and mechanical competence. These axes are not sequential interventions but coordinated regulatory dimensions whose alignment determines long term tissue stability. The right panel depicts the stabilized tissue state achieved when redox balance and biomechanical maturation converge. Maintenance of an optimal redox window sustains cellular viability and ECM synthesis, while increased crosslink density establishes load bearing structural integrity. Their coordinated operation defines a mature three-dimensional tissue architecture capable of withstanding diffusion constraints and mechanical stress.

### 1.1 Scope and critical perspective

This review provides a critical and integrative evaluation of nanozyme- and immobilized enzyme-based strategies for scaffold-free cell sheet engineering. Rather than simply summarizing recent advances, published studies are systematically synthesized and critically evaluated with respect to their experimental design, catalytic mechanisms, biological outcomes, translational relevance, current limitations, and remaining knowledge gaps. Throughout the manuscript, experimentally validated evidence is clearly distinguished from conceptual hypotheses, theoretical assumptions, and future research perspectives, thereby providing a balanced and evidence-based interpretation of the current state of the field.

Building upon this critical synthesis, we propose the Reciprocal Dual-Axis Catalytic Framework as a conceptual model that integrates existing evidence while identifying unresolved challenges and future research opportunities. Rather than treating nanozymes and immobilized enzymes as independent technologies, the proposed framework critically examines their complementary roles, current limitations, and opportunities for integration within scaffold-free cell sheet engineering. Accordingly, each major section systematically synthesizes and critically evaluates published studies to identify established evidence, remaining knowledge gaps, and future research directions relevant to scaffold-free cell sheet engineering. To the best of our knowledge, based on our critical evaluation of the available literature, although previous reviews have separately discussed nanozymes, extracellular matrix engineering, and cell sheet engineering, no review has systematically examined the complementary integration of nanozyme-mediated redox regulation and immobilized enzyme-mediated structural catalysis within the specific context of scaffold-free cell sheet engineering.

## 2 Biomedical challenges in cell sheet engineering: hypoxia, ROS, and ECM instability

In scaffold-free cell sheets, structural integrity is maintained primarily through cell generated extracellular matrix (ECM) and intercellular junctions in the absence of an external supporting framework (Owaki et al., 2014). While this structural autonomy preserves native cell-matrix interactions, it also renders the construct highly sensitive to perturbations in its internal microenvironment (Hu D. et al., 2023; Lin et al., 2025). As cell sheets mature through three-dimensional stacking, diffusion limitations of oxygen and nutrients become progressively unavoidable (Gholipourmalekabadi et al., 2016; Khodamoradi et al., 2026; Carmelite and Jain, 2000). These diffusion constraints extend beyond simple metabolic restriction, triggering hypoxia induced metabolic reprogramming, enhances reactive oxygen species (ROS) accumulation, and disrupts ECM remodeling processes (Hamanaka and Chandel, 2009). Collectively, these alterations progressively compromise interact to form a coupled network of molecular perturbations that progressively undermine matrix integrity, indicating that the structural stability of scaffold-free cell sheets is governed not only by architectural organization but also by tightly coupled biochemical and biomechanical processes.

Although the individual roles of hypoxia, oxidative stress, ECM remodeling, and mechanical instability have been extensively investigated, they have largely been studied as independent biological events. Consequently, current understanding remains fragmented, and the reciprocal interactions between biochemical regulation and structural maturation are insufficiently integrated within the context of scaffold-free cell sheet engineering. This limitation highlights an important knowledge gap in the field and underscores the need for a systems-level perspective that links these interconnected pathological processes. The following sections therefore critically examine the experimental evidence underlying each component of this failure network before integrating them into the conceptual framework proposed in this review.

### 2.1 Hypoxia-induced metabolic reprogramming and cell viability

Within multilayered cell sheets, oxygen diffusion becomes intrinsically limited, and localized hypoxic regions emerge once tissue thickness exceeds approximately 100–200 μm (Carmelite and Jain, 2000; Matsuura et al., 2014). These diffusion constraints extend beyond reductions in oxygen availability and act as a molecular inflection point that reshapes cellular metabolic programs. Under hypoxic conditions, hypoxia inducible factor 1 alpha, HIF 1α, becomes stabilized and drives a metabolic shift toward glycolysis, accompanied by reduced mitochondrial oxidative phosphorylation efficiency (Hamanaka and Chandel, 2009). This metabolic transition leads to diminished ATP production, lactate accumulation, and intracellular acidification, all of which directly influence the energetic demands of extracellular matrix synthesis and protein stability (Wu et al., 2023). Concurrently, increased electron leakage from mitochondrial electron transport chain complexes I and III enhances superoxide generation, thereby mechanistically linking hypoxia to reactive oxygen species accumulation (Bleier and Dröse, 2013). In this context, hypoxia should not be regarded merely as oxygen deprivation, but as an upstream regulator that amplifies redox imbalance (Hamanaka and Chandel, 2009).

Moreover, HIF 1α regulates not only vascular endothelial growth factor but also specific matrix metalloproteinases, indicating a direct intersection between hypoxic signaling and extracellular matrix remodeling pathways (González-Ávila et al., 2023). The convergence of metabolic reprogramming and transcriptional regulation compromises both cell viability and matrix synthesis, thereby establishing an early molecular link between metabolic stress and structural instability in scaffold-free cell sheets.

Collectively, these findings indicate that diffusion-limited hypoxia represents the earliest molecular event driving progressive dysfunction in scaffold-free cell sheets. However, current evidence has largely focused on metabolic adaptation and cell survival, whereas the direct contribution of hypoxia to long-term ECM maturation and tissue mechanics remains comparatively underexplored. Consequently, hypoxia should be viewed not only as a metabolic stressor but also as a critical upstream regulator linking metabolic reprogramming to subsequent structural deterioration.

### 2.2 ROS-mediated oxidative damage to the ECM network

Excessive accumulation of reactive oxygen species (ROS) is a major driver of structural destabilization in scaffold-free constructs. Elevated levels of superoxide and hydrogen peroxide promote lipid peroxidation and oxidative modification of proteins (Wang et al., 2023). Major structural proteins such as collagen are particularly susceptible to oxidative disruption of triple helix stability and fragmentation (Brown et al., 2015), which compromises the molecular continuity of the extracellular matrix network (Martins et al., 2021). Beyond direct oxidative injury, ROS interferes with enzyme mediated crosslinks and promotes aberrant non enzymatic crosslink formation, thereby perturbing fibrillar alignment and mechanical homogeneity within the matrix (Lloyd and He, 2024). Sustained oxidative stress also promotes the accumulation of advanced glycation end products and thiol oxidation, further reducing matrix elasticity and resilience (Lyu et al., 2023). Collectively, these molecular alterations progressively impair the structural continuity, elasticity, and mechanical resilience of the extracellular matrix, thereby compromising the intrinsic stability of scaffold-free cell sheets.

In addition, ROS activates redox sensitive signaling pathways, including NF kappa B, leading to upregulation of matrix metalloproteinases and enhanced protease mediated matrix degradation. When direct molecular damage coincides with proteolytic remodeling, crosslink density progressively declines, resulting in reduced tensile resistance and compromised structural integrity in scaffold-free tissues (Ma et al., 2023). In scaffold-free systems lacking external mechanical support, oxidative alterations directly translate into progressive loss of tissue integrity and mechanical competence (Cooper and Rainbow, 2022).

Taken together, available evidence indicates that ROS-mediated oxidative stress extends beyond molecular damage to become a central determinant of ECM destabilization in scaffold-free constructs. Nevertheless, most studies have primarily characterized oxidative injury itself, whereas the quantitative relationship between ROS regulation, matrix remodeling, and long-term mechanical stabilization remains insufficiently defined. Addressing this gap will be essential for the rational design of catalytic strategies capable of preserving both redox homeostasis and structural integrity in scaffold-free cell sheets.

### 2.3 Imbalance of protease activity and structural delamination

Extracellular matrix homeostasis is maintained through a finely regulated balance between synthesis and degradation, commonly described as matrix turnover (Fu et al., 2024). In scaffold-free cell sheets exposed to hypoxia and elevated ROS, expression of matrix degrading enzymes such as MMP 2 and MMP 9 increases, while the regulatory balance of tissue inhibitors of metalloproteinases, TIMPs, becomes disrupted (González-Ávila et al., 2023; Campo et al., 2013). Alterations in the MMP-to- TIMP ratio shift ECM turnover toward a degradation-dominant state, creating conditions in which matrix breakdown exceeds matrix synthesis (Rainero, 2016). Sustained degradation of collagen and basement membrane components weakens the continuity of the ECM network and disrupts established load transfer pathways within the construct (Martins et al., 2021). In scaffold-free systems, where no external framework compensates for matrix loss, even moderate reductions in ECM density translate directly into mechanical vulnerability. This effect is particularly pronounced at the interfaces between stacked cell sheets, where even modest reductions in matrix connectivity generate stress concentrations, weaken interlayer cohesion, and increase susceptibility to delamination (Cooper and Rainbow, 2022; Okuda and Fujimoto, 2020).

Thus, even subtle imbalances in protease activity are amplified in scaffold-free constructs, extending beyond localized matrix degradation to compromise three dimensional integration and overall mechanical stability. Rather than representing an isolated molecular disturbance, such dysregulation marks the transition by which biochemical imbalance becomes translated into tissue level structural instability (Zhang et al., 2026; Papafilippou et al., 2025).

Taken together, available evidence indicates that protease dysregulation represents a critical step linking oxidative matrix damage to tissue-level structural failure in scaffold-free cell sheets. However, relatively few studies have directly examined how altered protease activity influences long-term mechanical maturation and interlayer cohesion in multilayer constructs. Addressing this gap will be important for developing catalytic strategies capable of preserving ECM integrity while maintaining structural stability during tissue maturation.

### 2.4 Insufficient mechanical reinforcement and crosslink density

Mechanical strength of cell sheets depends not only on collagen deposition but also on the density and organization of enzyme-mediated crosslinks, particularly those regulated by lysyl oxidase (LOX). LOX oxidizes lysine residues in collagen and elastin to generate reactive aldehydes that initiate covalent crosslink formation, thereby enhancing interfibrillar bonding and improving tensile strength and fatigue resistance (Lloyd and He, 2024; Elbjeirami et al., 2003; Makris et al., 2013). Hypoxic stress and metabolic imbalance impair LOX expression and enzymatic activity, resulting in reduced or heterogeneous crosslink formation. Consequently, diminished crosslink density compromises ECM load distribution and mechanical resilience, rendering scaffold-free cell sheets particularly vulnerable to structural failure because no external framework compensates for endogenous mechanical deficiencies (Makris et al., 2013; Cooper and Rainbow, 2022; Elbjeirami et al., 2003).

Beyond its structural role, crosslink density also regulates cellular mechanotransduction through changes in matrix stiffness (Zhao et al., 2020). Reduced matrix stiffness alters cytoskeletal organization, YAP/TAZ signaling, and downstream transcriptional programs involved in ECM synthesis and remodeling (Dupont et al., 2011). Unlike scaffold-based constructs, scaffold-free cell sheets rely almost entirely on endogenous crosslink formation to achieve mechanical maturation. Consequently, adequate crosslink density is not only a structural requirement but also a key regulator of tissue maturation and long-term mechanical stability (Cooper and Rainbow, 2022; Elbjeirami et al., 2003).

From a systems perspective, the evidence discussed in Sections 2.1–2.4 indicates that hypoxia, ROS accumulation, ECM degradation, protease dysregulation, and reduced crosslink density are not independent pathological events. Rather, they represent interconnected processes that collectively determine the structural integrity of scaffold-free cell sheets. Hypoxia-induced metabolic reprogramming promotes ROS generation, which subsequently drives ECM oxidation, protease activation, and progressive matrix destabilization (Hamanaka and Chandel, 2009; Wang et al., 2023; Martins et al., 2021; Ma et al., 2023). Concurrently, reduced crosslink density compromises load-bearing capacity and mechanical resilience, while altered matrix integrity further influences mechanotransduction and cellular responses (Elbjeirami et al., 2003; Zhao et al., 2020; Dupont et al., 2011). Taken together, these findings support the concept that biochemical imbalance and biomechanical insufficiency are coupled through reciprocal interactions rather than a simple linear cascade. Although substantial experimental evidence supports each individual component of this failure network, direct validation of their reciprocal interactions within scaffold-free cell sheet systems remains limited. To facilitate this systems-level perspective, Table 1 summarizes the major molecular events, microenvironmental alterations, structural consequences, and potential catalytic intervention strategies. Building upon this synthesis, the Reciprocal Dual-Axis Catalytic Framework proposed in this review should be regarded as a conceptual integration of existing evidence that highlights important knowledge gaps and provides a systems-level rationale for future investigation rather than a fully validated mechanistic model.

**TABLE 1:** Summary of interconnected molecular failure pathways and potential catalytic intervention opportunities in scaffold-free cell sheets.

Challenge	Key molecular events	Microenvironmental shift	Structural consequence	Potential catalytic intervention strategies
Hypoxia	HIF 1α stabilization, LDH upregulation, mitochondrial complex I and III electron leakage	Glycolytic shift, lactate accumulation, ROS amplification	Reduced ECM synthesis, decreased cell viability	Redox axis – Potential oxygen-generating nanozymes (e.g., MnO2 -based catalytic systems)
ROS burst	Superoxide and hydrogen peroxide accumulation, lipid peroxidation, NF kappa B signaling activation	Protein oxidation, crosslink destabilization, AGEs accumulation	Collagen denaturation, reduced crosslink density, mechanical weakening	Redox axis – Potential SOD- and catalase-like nanozymes (e.g., CeO2-based catalytic systems)
ECM instability	MMP 2 and MMP 9 upregulation, TIMP imbalance	Accelerated ECM turnover, degradation greater than synthesis	Delamination, loss of structural integration	Structural axis – Potential immobilized enzyme systems for protease regulation
Mechanical weakness	Reduced LOX mediated crosslinking, fibril misalignment	Decreased covalent crosslink density	Lower tensile modulus, reduced shear resistance	Structural axis – Potential crosslink-promoting immobilized enzymes (e.g., immobilized lysyl oxidase and transglutaminase

^a^ This table summarizes representative pathological events and potential catalytic intervention opportunities synthesized from the current literature. The proposed intervention strategies represent conceptual design opportunities synthesized from the current literature and should not be interpreted as experimentally validated therapeutic recommendations.

## 3 Nanozymes for ECM modulation and cell sheet viability

As discussed in Section 2, structural stability in scaffold-free cell sheets is governed by a dynamic equilibrium between redox homeostasis and extracellular matrix (ECM) integrity. Among the various catalytic strategies proposed to regulate this microenvironment, nanozymes have attracted increasing attention because of their enzyme-mimetic catalytic activities and sustained redox regulatory capacity (Shen et al., 2025). Rather than functioning merely as antioxidant adjuncts, nanozymes can actively modulate the biochemical microenvironment by regulating reactive oxygen species levels under diffusion-limited conditions.

During progressive cell sheet thickening, oxygen diffusion becomes increasingly restricted, leading to localized hypoxia, mitochondrial dysfunction, and excessive reactive oxygen species accumulation, as demonstrated in various three-dimensional tissue models (Hamanaka and Chandel, 2009; Bettahalli et al., 2011). These pathological alterations provide a mechanistic rationale for applying catalytic redox regulation to scaffold-free cell sheet engineering. However, because most current evidence has been generated in tumor, inflammatory, or wound-healing models rather than scaffold-free cell sheet, careful interpretation is warranted when translating these findings into the unique physicochemical environment of multilayered cell sheet constructs.

Accordingly, this section critically evaluates representative nanozyme systems with respect to their catalytic mechanisms, experimental evidence, current limitations, and potential relevance to scaffold-free cell sheet engineering. Particular emphasis is placed on how nanozyme-mediated redox regulation may preserve cell viability and extracellular matrix integrity while highlighting important knowledge gaps that remain to be addressed before broader implementation in scaffold-free cell sheet engineering.

### 3.1 Representative nanozyme systems for redox regulation

Diffusion-limited oxygen transport and increased cellular density expose scaffold-free cell sheets to persistent oxidative stress, making regulation of the local redox microenvironment an important objective for maintaining tissue integrity. Among the various catalytic approaches proposed for redox regulation, nanozymes have attracted considerable attention because their enzyme-mimetic catalytic activities enable repeated ROS modulation through catalytic turnover rather than stoichiometric consumption (Shen et al., 2025). Unlike natural enzymes, nanozymes exhibit high catalytic stability, tunable physicochemical properties, and resistance to denaturation, making them attractive candidates for sustained redox regulation under diffusion-limited conditions. Consequently, nanozymes are increasingly recognized as promising catalytic platforms for preserving redox homeostasis in diffusion-limited three-dimensional tissue systems.

ROS concentrations within thick cell sheets differ substantially from those observed under conventional two-dimensional culture conditions due to diffusion constraints and increased cellular density. In three-dimensional tissue models characterized by pronounced diffusion limitation, intracellular ROS levels rise beyond basal low nanomolar ranges and can reach tens to hundreds of nanomolar concentrations (Sies, 2017; Huang and Sikes, 2014; Chen L. et al., 2025). Such elevations exceed the physiological signaling window of ROS and approach thresholds associated with oxidative modification of structural proteins and perturbation of extracellular matrix organization. Although direct quantitative measurements in scaffold-free cell sheets remain limited, diffusion-limited multilayered constructs provide a mechanistic basis for localized ROS accumulation. Under these conditions, redox regulation should be considered not simply as antioxidant supplementation but as a quantitative design strategy that balances ROS production with catalytic turnover capacity while maintaining a functional redox window (Shen et al., 2025; Bettahalli et al., 2011; Huang and Sikes, 2014).

Representative nanozyme systems employ distinct catalytic mechanisms to regulate oxidative stress. CeO_2_-based nanozymes undergo reversible Ce^3+^/Ce^4+^ redox cycling, enabling repeated scavenging of superoxide and hydrogen peroxide through combined superoxide dismutase- and catalase-like activities (Shen et al., 2025; Sre et al., 2025). In contrast, MnO_2_-based nanozymes primarily catalyze hydrogen peroxide decomposition while simultaneously increasing local oxygen availability, thereby alleviating diffusion-induced hypoxia (Camci-Unal et al., 2013; Shen et al., 2025). Compared with conventional small-molecule antioxidants, these catalytic properties provide the potential for prolonged redox regulation under diffusion-limited conditions because catalytic activity is maintained through repeated turnover rather than one-time chemical consumption.

To facilitate systematic comparison of representative nanozyme systems relevant to scaffold-free cell sheet engineering, Table 2 compares their catalytic mechanisms, primary biological functions, representative biomedical applications, current experimental evidence, translational challenges, and representative supporting references. Rather than serving as a descriptive summary alone, the table critically evaluates the current level of evidence and translational potential of each nanozyme platform. This comparison illustrates that while CeO_2_- and MnO_2_-based nanozymes currently represent the most promising catalytic platforms for redox regulation, direct evidence supporting their application in scaffold-free cell sheet engineering remains limited, highlighting an important knowledge gap for future investigation.

**TABLE 2:** Representative nanozyme systems relevant to scaffold-free cell sheet engineering, highlighting catalytic mechanisms, biological functions, current evidence, potential advantages, translational limitations, and representative supporting references.

Nanozyme	Catalytic mechanism	Primary biological effect	Representative biomedical applications	Potential advantages for scaffold-free cell sheets	Evidence in scaffold-free cell sheets	Safety and translational challenges	References
CeO_2_	SOD-like + catalase-like	ROS scavenging	Cardiac, skin, wound healing	Sustained ROS regulation, preservation of ECM	Limited direct evidence	Limited scaffold-free validation; long-term biocompatibility requires further investigation	Shen et al., 2025; Sre et al., 2025
MnO_2_	Catalase-like, oxygen generation	Hypoxia alleviation	Ischemic tissues, tumor models	Oxygen supplementation under diffusion limitation	Indirect evidence	Performance depends on local H2O2; biosafety requires validation	Camci-Unal et al., 2013; Shen et al., 2025
Fe-based nanozymes	Peroxidase-like	ROS modulation	Cancer therapy	Tunable catalytic activity	No direct evidence	Potential oxidative side effects; limited scaffold-free validation	Shen et al. (2025)
Pt-based nanozymes	Catalase-like	ROS decomposition	Ischemic injury	High catalytic stability	No direct evidence	High cost; limited translational scalability	Shen et al. (2025)
Multi-enzyme nanozymes	Multiple enzyme-mimetic activities	Integrated redox regulation	Complex tissue engineering	Multifunctional catalytic regulation	Emerging direct evidence	Limited long-term evaluation and translational validation	Shen et al. (2025)

As summarized in Table 2, current evidence supporting nanozyme-mediated redox regulation has been derived predominantly from tumor, inflammatory, ischemic, and wound-healing models rather than scaffold-free cell sheet systems. Furthermore, nanozyme catalytic performance is influenced by local oxygen availability, ROS concentration, pH, substrate accessibility, and tissue architecture, all of which differ substantially in densely stacked scaffold-free cell sheets. Consequently, although current findings provide a strong mechanistic rationale for applying nanozymes to scaffold-free cell sheet engineering, their therapeutic performance should be interpreted cautiously until validated under diffusion-limited cell sheet conditions. Future studies should therefore prioritize quantitative validation of nanozyme-mediated redox regulation directly within scaffold-free cell sheet models while simultaneously addressing long-term biosafety, biocompatibility, and translational feasibility.

### 3.2 Redox-sensitive modulation of the ECM microenvironment

Reactive oxygen species (ROS) should not be regarded solely as cytotoxic byproducts but also as essential regulators of extracellular matrix (ECM) remodeling and tissue homeostasis. Moderate ROS levels participate in physiological signaling pathways that regulate matrix metalloproteinase expression, cell differentiation, mechanotransduction, and ECM remodeling through redox sensitive signaling pathways including NF-κB and MAPK (González-Ávila et al., 2023; Ma et al., 2023). However, excessive ROS accumulation shifts these regulatory pathways toward pathological activation, promoting oxidative damage to structural proteins together with protease-mediated matrix degradation. Consequently, elevated ROS levels simultaneously accelerate oxidative modification of ECM components and abnormal matrix turnover, thereby establishing two complementary mechanisms of ECM destabilization. (Wang et al., 2023; Ma et al., 2023; Shen et al., 2025).

Collectively, these findings indicate that the objective of nanozyme-mediated redox regulation should not be complete ROS elimination but the maintenance of a functional redox window that preserves physiological signaling while preventing oxidative amplification. Within this optimal range, ROS-dependent signaling required for cell differentiation, mechanotransduction, and tissue maturation is maintained, whereas pathological oxidative stress is constrained (Dupont et al., 2011; Sies, 2017). Accordingly, nanozyme design should prioritize balanced modulation of ROS rather than maximal antioxidant activity.

Nevertheless, the quantitative boundaries defining this functional redox window remain poorly established, particularly in diffusion-limited scaffold-free cell sheet systems. Most available evidence has been derived from conventional cell culture or other three-dimensional tissue models, wheareas direct quantitative evaluation of ROS thresholds that support long-term ECM maturation and mechanical stability in scaffold-free cell sheets remains limited. Therefore, defining the quantitative boundaries of this functional redox window represents an important knowledge gap and an essential prerequisite for the rational design of nanozyme-based catalytic strategies in scaffold-free tissue engineering.

### 3.3 Opportunities, limitations, and translational considerations of nanozyme-based strategies

Nanozymes offer several advantages for regulating oxidative stress and preserving extracellular matrix homeostasis in scaffold-free cell sheet engineering. Their catalytic activities have shown considerable promise across regenerative applications, including cardiac, cartilage, skin, and corneal tissue engineering, where preservation of cellular viability and extracellular matrix stability is essential for functional tissue maturation (Homma et al., 2020; Lin et al., 2025; Rasouli et al., 2023). Their catalytic turnover enables sustained modulation of the redox microenvironment without rapid depletion, making them particularly attractive for diffusion-limited three-dimensional constructs. When interpreted within the hypoxia–ROS–ECM failure network proposed in Section 2, nanozymes function primarily as upstream catalytic regulators that suppress excessive ROS amplification before oxidative damage propagates to extracellular matrix oxidation and protease activation (Hamanaka and Chandel, 2009; Ma et al., 2023; Shen et al., 2025; Sre et al., 2025). By preserving redox homeostasis, nanozymes help maintain cellular viability and create a biochemical microenvironment that supports extracellular matrix synthesis and long-term tissue stability.

Despite these advantages, several important limitations should be considered. Most experimental evidence supporting nanozyme-mediated redox regulation has been generated in tumor, inflammatory, ischemic, or wound-healing models rather than scaffold-free cell sheet systems (Zhou et al., 2025; Hu S. et al., 2023; Shen et al., 2025). Furthermore, nanozyme performance is likely influenced by oxygen availability, ROS concentration, pH, substrate accessibility, and tissue architecture, all of which differ substantially in densely stacked scaffold-free constructs. In addition, important translational challenges including nanoparticle biocompatibility, long-term tissue accumulation, biodegradation, manufacturing scalability, and regulatory considerations remain insufficiently investigated for scaffold-free tissue engineering applications.

Importantly, restoration of redox balance alone cannot reconstruct diminished crosslink density or recover mechanical competence once structural deterioration has occurred (Martins et al., 2021; Elbjeirami et al., 2003; Makris et al., 2013; Cooper and Rainbow, 2022). Thus, although nanozymes preserve a permissive biochemical microenvironment, they do not directly restore extracellular matrix architecture. These limitations indicate that long-term stabilization of scaffold-free cell sheets requires complementary catalytic strategies that directly reinforce extracellular matrix structure. Accordingly, the following section examines immobilized enzyme-based structural catalysis as a complementary strategy for directly reinforcing extracellular matrix architecture within the proposed Reciprocal Dual-Axis Catalytic Framework.

## 4 Complementary catalytic strategies for biomechanical stabilization of scaffold-free cell sheets

As established in Section 3, the stability of scaffold-free cell sheets depends on the maintenance of redox homeostasis. Catalytic redox modulation *via* nanozymes operates at upstream nodes of the hypoxia ROS cascade, limiting oxidative amplification and establishing a permissive biochemical environment that supports cellular viability and matrix preservation. However, most catalytic strategies developed to date have primarily focused on restoring biochemical homeostasis, whereas comparatively little attention has been directed toward reconstruction of the endogenous mechanical architecture. Consequently, restoration of redox balance alone does not necessarily translate into durable biomechanical competence in scaffold-free tissues.

In scaffold-free systems, where no external framework exists, the extracellular matrix constitutes the primary load bearing architecture (Zhao et al., 2020; Holzapfel et al., 2025). Although enzyme-mediated crosslinking has been extensively investigated in biomaterials and hydrogel engineering, comparatively little attention has been directed toward its biomechanical implications in scaffold-free tissues, where the endogenous ECM must function as the sole load-bearing architecture. Crosslink density, fiber alignment, and resistance to tensile and shear stresses therefore emerge as decisive determinants of structural maturity (Papafilippou et al., 2025; Makris et al., 2013). Mechanical functions typically provided by synthetic scaffolds in conventional tissue engineering must be assumed entirely by the endogenous ECM network. Within this context, enzyme mediated crosslinking should be understood not merely as a molecular modification, but as structural catalysis that reorganizes ECM architecture at the network level. Biochemical instability and biomechanical insufficiency are tightly coupled in thick constructs. Diffusion limitation, redox amplification, protease dysregulation, and crosslink loss interact through a reaction-diffusion-mechanics network that links molecular imbalance to progressive mechanical weakening. Under diffusion constrained conditions, this coupling forms a self-reinforcing failure architecture.

**FIGURE 2:**
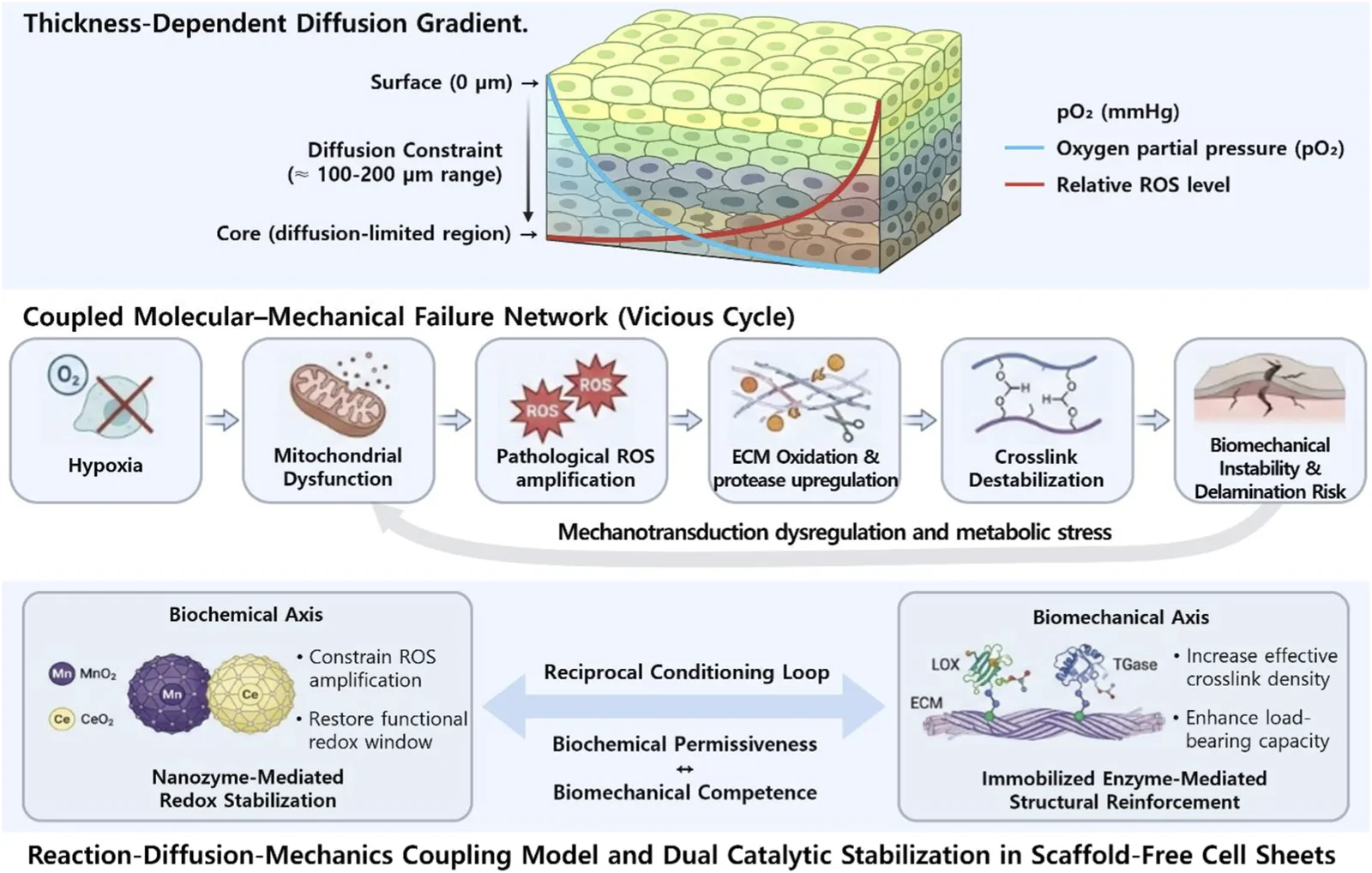
Reaction–diffusion–mechanics coupling model illustrating the interconnected failure network in thick scaffold-free cell sheets and the reciprocal dual-axis catalytic stabilization strategy. The model summarizes current evidence and highlights the proposed complementary roles of nanozyme-mediated redox regulation and immobilized enzyme-mediated structural reinforcement.

Figure 2 summarizes the systems-level interpretation developed throughout Sections 2, 3. Rather than depicting independent molecular pathways, the framework integrates biochemical instability and biomechanical insufficiency into a coupled reaction–diffusion–mechanics network. This perspective provides the conceptual rationale for introducing immobilized enzyme-mediated structural catalysis as a complementary regulatory axis. In this systems-level formulation, redox catalysis secures biochemical permissiveness, whereas structural catalysis establishes biomechanical competence. These dimensions operate in parallel and condition one another: structural maturation depends on redox stabilization, and restored mechanical integrity reshapes mechanotransduction and metabolic feedback. Alignment of these axes enables scaffold-free tissues to withstand diffusion constraints and mechanical loading simultaneously. In dense constructs, effective structural catalysis further requires spatial control and sustained enzymatic activity. This requirement exposes limitations of freely diffusing enzymes and motivates immobilization as a strategy for spatially confined catalytic reinforcement (Yu et al., 2026; Agrawal et al., 2008). The following sections critically examine representative immobilized enzyme strategies, their mechanistic basis, current limitations, and opportunities for integration within the proposed Reciprocal Dual-Axis Catalytic Framework.

### 4.1 Structural catalysis in ECM reinforcement: a parallel axis to redox regulation

As discussed in Section 3, stabilization of scaffold-free cell sheets initially depends on restoration of redox homeostasis. Nanozyme-mediated redox catalysis operates at upstream nodes of the hypoxia-ROS cascade, limiting oxidative amplification and establishing a permissive biochemical environment that supports cellular viability and extracellular matrix (ECM) synthesis. However, restoration of redox balance alone does not necessarily translate into long-term structural maturation (Lloyd and He, 2024; Makris et al., 2013). Once crosslink density and network connectivity have deteriorated, mechanical competence cannot be fully restored solely through regulation of oxidative stress. These limitations highlight the need for complementary catalytic strategies that directly reinforce the structural architecture of scaffold-free tissues.

This requirement is particularly important in scaffold-free constructs, where the endogenous ECM functions as the sole load-bearing architecture in the absence of an external supporting scaffold (Zhang et al., 2026; Zhao et al., 2020; Holzapfel et al., 2025). Mechanical integrity therefore depends not only on matrix deposition but also on the organization of the ECM network, including crosslink density, fibrillar connectivity, and collagen alignment, which collectively determine tensile strength, shear resistance, and overall structural stability (Papafilippou et al., 2025; Elbjeirami et al., 2003; Makris et al., 2013; Kuth and Boccaccini, 2024). From this perspective, enzyme-mediated crosslinking should be regarded not merely as a molecular modification but as structural catalysis that directly reinforces the mechanical architecture of scaffold-free tissues. Moreover, ECM stiffness and crosslink organization regulate mechanotransduction pathways that influence cytoskeletal organization, cell behavior, and matrix remodeling, indicating that structural catalysis contributes not only to mechanical reinforcement but also to long-term tissue maturation (Dupont et al., 2011; Shen et al., 2025; Yi et al., 2022).

Although endogenous ECM assembly contributes to tissue development, spontaneous matrix remodeling is often insufficient to establish uniform mechanical competence in dense or multilayered scaffold-free constructs, particularly under diffusion-limited conditions (Makris et al., 2013; Bettahalli et al., 2011; Efremov et al., 2021). Furthermore, freely diffusing enzymes exhibit limited penetration and heterogeneous catalytic activity within thick tissues, leading to spatially non-uniform crosslink formation and incomplete structural reinforcement (Bettahalli et al., 2011; Ariaeenejad et al., 2026; Guvendiren et al., 2010). Collectively, these limitations provide the rationale for introducing immobilized enzyme-mediated catalytic reinforcement as a complementary strategy to endogenous ECM maturation. Rather than replacing intrinsic tissue formation, structural catalysis is intended to augment crosslink formation, improve network organization, and promote long-term biomechanical stability. The following sections therefore critically examine representative immobilized enzyme systems, emphasizing their catalytic mechanisms, current evidence, translational challenges, and potential integration within the proposed Reciprocal Dual-Axis Catalytic Framework.

### 4.2 Enzyme-mediated crosslinking and its Impact on tensile and shear integrity in ECM architecture

The structural significance of enzyme-mediated crosslinking extends beyond molecular bond formation to the regulation of tissue-level mechanical behavior. In scaffold-free cell sheets, where the extracellular matrix (ECM) serves as the primary load-bearing architecture, crosslinking reactions function as structural engineering processes that reorganize load-transfer pathways throughout the matrix network rather than merely introducing additional chemical bonds (Zhao et al., 2020). Consequently, structural catalysis should be interpreted in terms of its ability to improve mechanical integration, enhance stress distribution, and reinforce long-term tissue stability.

Lysyl oxidase (LOX) catalyzes oxidative deamination of lysine residues in collagen and elastin to generate reactive allysine intermediates that subsequently form covalent intermolecular crosslinks (Kumari et al., 2016). Increased crosslink density restricts relative fibrillar sliding, enhance network connectivity, and improves homogeneous stress transfer during mechanical loading. Accordingly, increases in LOX-mediated crosslink formation correlate with enhanced tensile stiffness, Young’s modulus, fatigue resistance, and reduced creep under sustained loading (Elbjeirami et al., 2003; Marturano et al., 2014; Fessel and Snedeker, 2009; Salvatore et al., 2021). These mechanical properties are particularly important for scaffold-free tissues exposed to repetitive physiological loading, such as cardiac pulsation or continuous tissue deformation. Collectively, current evidence consistently supports LOX as a principal regulator of collagen maturation and tensile reinforcement. Nevertheless, most available studies have been conducted in native connective tissues or engineered biomaterials, whereas direct validation of LOX-mediated structural reinforcement in scaffold-free cell sheet systems remains limited.

In contrast, transglutaminase (TGase) catalyzes formation of ε-(γ-glutamyl)-lysine isopeptide bonds between glutamine and lysine residues across multiple ECM proteins, including collagen, fibronectin, and laminin (Selcuk et al., 2024). Rather than primarily increasing collagen maturation, TGase strengthens interprotein connectivity and improves three-dimensional matrix cohesion. Consequently, TGase-mediated crosslinking contributes predominantly to shear resistance by reducing interfacial sliding and maintaining matrix continuity under multidirectional mechanical loading (Salvatore et al., 2021; Selcuk et al., 2024). Compared with LOX, TGase has received comparatively less attention in scaffold-free tissue engineering. Although existing studies demonstrate improved matrix cohesion and shear stability, quantitative evaluation of its long-term contribution to tissue-scale mechanical maturation remains limited.

Beyond increasing matrix stiffness, enzyme-mediated crosslinks regulate energy dissipation, resistance, and interfacial stability at the network level. Well-integrated crosslinked matrices distribute mechanical energy through controlled microdeformation, thereby delaying crack propagation and enhancing structural resilience (Svensson et al., 2013; Vanderheiden et al., 2015). Conversely, heterogeneous crosslink distribution promotes local stress concentration and increases susceptibility to interfacial failure, particularly within multilayered scaffold-free cell constructs where stacked interfaces represent mechanically vulnerable regions (Hong et al., 2017). Enzyme-mediated crosslinking therefore shifts failure behavior from adhesive interfacial separation toward cohesive internal fracture while simultaneously modulating mechanotransduction through focal adhesion assembly, cytoskeletal tension, and YAP/TAZ signaling (Dupont et al., 2011; Yi et al., 2022).

Taken together, current evidence suggests that LOX and TGase reinforce extracellular matrix architecture through complementary rather than identical mechanisms. LOX primarily promotes collagen maturation and tensile reinforcement, whereas TGase predominantly enhances interprotein cohesion and shear stability. Despite these promising findings, direct comparative studies in scaffold-free cell sheet systems remain scarce, limiting evidence-based selection of enzyme systems for specific tissue engineering applications. To facilitate systematic comparison of representative immobilized enzyme systems, Table 3 summarizes their catalytic mechanisms, mechanical functions, current experimental evidence, translational limitations, and representative supporting references.

**TABLE 3:** Representative immobilized enzyme systems relevant to scaffold-free cell sheet engineering, comparing catalytic mechanisms, primary mechanical functions, current experimental evidence, translational challenges, and representative supporting references. Evidence categories distinguish direct experimental validation in scaffold-free cell sheet systems from indirect evidence derived from related tissue engineering models.

Immobilized enzyme system	Catalytic mechanism	Primary mechanical function	Representative tissue engineering applications	Potential advantages for scaffold-free cell sheets	Evidence in scaffold-free cell sheets	Safety and translational challenges	References
Lysyl oxidase (LOX)	Oxidative deamination of lysine residues to generate covalent collagen/elastin crosslinks	Tensile reinforcement, collagen maturation, fatigue resistance	Tendon, ligament, skin, vascular tissue engineering	Improves collagen organization, tensile strength, and long-term mechanical stability	Limited direct evidence	Difficult spatial control; limited scaffold-free validation; enzyme immobilization required	Kumari et al., 2016; Elbjeirami et al., 2003; Marturano et al., 2014; Makris et al., 2013
Transglutaminase (TGase)	Formation of ε-(γ-glutamyl)-lysine isopeptide bonds between ECM proteins	Matrix cohesion, shear resistance, interfacial stabilization	Cartilage, skin, wound healing	Enhances interprotein connectivity and interlayer cohesion	Indirect evidence	Limited quantitative evaluation of long-term tissue maturation	Selcuk et al., 2024; Salvatore et al., 2021
Tyrosinase	Oxidative coupling of phenolic residues to generate dityrosine crosslinks	Surface stabilization and ECM strengthening	Hydrogel biofabrication, tissue adhesives	Enables mild enzymatic crosslinking under physiological conditions	No direct evidence	Limited application to scaffold-free tissues	Ariaeenejad et al. (2026)
Laccase	Oxidation of phenolic substrates leading to intermolecular crosslink formation	Matrix stabilization and network reinforcement	Hydrogel engineering, bioinks	Supports sustained enzymatic crosslinking under mild conditions	No direct evidence	Limited scaffold-free validation; enzyme stability considerations	Ariaeenejad et al. (2026)
Multi-enzyme immobilized systems	Combined catalytic crosslinking through spatially controlled immobilized enzyme platforms	Simultaneous mechanical reinforcement and prolonged catalytic activity	Advanced biomaterials, engineered ECM systems	Localized and sustained structural catalysis with improved catalytic persistence	Emerging proof-of-concept evidence	Manufacturing complexity; translational validation remains limited	Yu et al., 2026; Agrawal et al., 2008

### 4.3 Spatially confined catalysis: immobilization strategies for diffusion-limited scaffold-free systems

If enzyme mediated crosslinking provides the molecular basis for structural reinforcement, the spatial deployment of catalytic activity becomes an equally important design parameter in scaffold-free cell sheet engineering. In multilayered or thickened constructs, diffusion limitation governs the distribution of enzymes and substrates, causing catalytic reactions to become spatially heterogeneous rather than uniformly distributed throughout the tissue (Lovett et al., 2009; Griffith et al., 2005; McMurtrey, 2016). Consequently, the mechanical outcome of structural catalysis depends not only on enzymatic activity itself but also on the spatial distribution of catalytic reactions within the construct.

Freely diffusing enzymes preferentially initiate crosslinking near construct surfaces because concentration gradients limit penetration into diffusion-restricted interior regions (Agrawal et al., 2008; Ariaeenejad et al., 2026; Guvendiren et al., 2010). This phenomenon, commonly described as shell hardening, generates mechanically heterogeneous tissues characterized by highly crosslinked peripheral layers and relatively under-reinforced cores. Such heterogeneity promotes stress concentration, amplifies interfacial shear stress under mechanical loading, and increases susceptibility to crack initiation and delamination (Guvendiren et al., 2010; Vanderheiden et al., 2015; Bryant and Anseth, 2002; Cai et al., 2024; Benveniste and Miloh, 2001). Furthermore, freely diffusing enzymes exhibit limited residence time within dense tissues, restricting the sustained catalytic activity required for progressive ECM crosslink accumulation and long-term structural maturation (Ghajar et al., 2008; Ikeda-Imafuku et al., 2022).

Immobilization provides a fundamentally different strategy by transforming enzyme-mediated crosslinking into spatially controlled structural catalysis. Unlike freely diffusing enzymes, immobilized enzymes enable catalytic activity to be deliberately localized within mechanically strategic regions. However, immobilization itself does not inherently guarantee homogeneous catalytic activity throughout thick constructs. Variations in immobilization density, local substrate accessibility, and carrier material properties may still generate spatial heterogeneity, highlighting the importance of rational spatial design for achieving controlled catalytic distributions. Rather than serving solely to stabilize enzyme activity, immobilization enables catalytic reactions to be positioned deliberately at mechanically vulnerable regions, including sheet-sheet interfaces, diffusion-limited core regions, or predefined reinforcement zones. Localized catalytic reinforcement at interfacial regions promotes cross-sheet ECM continuity, improves shear transfer, enhances interlayer cohesion, and increases resistance to delamination (Ni et al., 2020; Barreiro et al., 2019). Likewise, targeted catalytic activity within diffusion-limited core regions has the potential to improve load distribution and reduce mechanical heterogeneity, although direct experimental validation in scaffold-free cell sheets remains limited (Ariaeenejad et al., 2026).

Beyond localized reinforcement, spatial control of immobilized enzyme distribution enables engineering of biomimetic mechanical gradients. In native tissues, gradual variations in stiffness and crosslink density facilitate stress redistribution and reduce abrupt mechanical failure. Analogously, gradient immobilization strategies may generate controlled stiffness transitions within scaffold-free constructs, thereby improving fracture resistance and mechanical resilience compared with uniformly crosslinked matrices (Qiao et al., 2025). In addition, immobilized enzymes maintain catalytic activity over extended periods, enabling progressive ECM crosslink accumulation and time-dependent mechanical maturation that more closely resembles physiological tissue development (Kutcherlapati et al., 2016). These characteristics distinguish immobilization from freely diffusing enzyme systems by providing both spatial precision and prolonged catalytic persistence.

Table 4 summarizes representative immobilization strategies relevant to scaffold-free cell sheet engineering, comparing their spatial design objectives, catalytic principles, expected mechanical benefits, current levels of experimental evidence, translational limitations, and representative supporting studies. Collectively, current evidence suggests that achieving precise spatial control of immobilized catalytic activity remains an important engineering challenge. Interface-targeted and long-term immobilization strategies show the greatest translational promise, whereas core-targeted and gradient immobilization remain largely conceptual approaches requiring experimental validation. Importantly, most available evidence has been derived from related biomaterials and tissue engineering systems rather than scaffold-free cell sheets, highlighting the need for direct validation of spatially controlled catalytic strategies in scaffold-free cell sheet engineering.

**TABLE 4:** Representative immobilization strategies for spatially controlled structural catalysis in scaffold-free cell sheet engineering, highlighting design objectives, current evidence, translational limitations, and supporting references.

Immobilization strategy	Spatial objective	Catalytic principle	Expected mechanical benefit	Current evidence	Major limitation	References
Surface immobilization	Reinforce peripheral ECM	Localized surface crosslinking	Improved surface stability	Indirect evidence	Shell hardening; poor interior penetration	Agrawal et al., 2008; Guvendiren et al., 2010
Interface immobilization	Strengthen sheet–sheet interfaces	Localized interfacial enzyme activity	Increased interlayer cohesion; reduced delamination	Emerging direct evidence	Limited validation in scaffold-free cell sheets	Ni et al., 2020; Barreiro et al., 2019
Core-targeted immobilization	Reinforce diffusion-limited regions	Localized catalytic activity within diffusion-limited core regions	Improved load distribution; reduced mechanical heterogeneity	No direct evidence (conceptual strategy)	Difficult enzyme delivery	Ariaeenejad et al. (2026)
Gradient immobilization	Mimic native stiffness gradients	Spatially controlled immobilized enzyme distribution	Reduced stress concentration; improved fracture resistance	No direct evidence (conceptual strategy)	Fabrication complexity	Qiao et al. (2025)
Long-term immobilization catalytic platforms	Sustain progressive ECM maturation	Persistent localized catalytic turnover	Progressive crosslink accumulation; prolonged mechanical maturation	Emerging direct evidence	Long-term in vivo validation lacking	Kutcherlapati et al., 2016; Yu et al., 2026

^a^ Evidence categories indicate the current level of experimental validation for scaffold-free cell sheet engineering. “Indirect evidence” refers to findings extrapolated from related tissue engineering models, whereas “Conceptual strategy” denotes engineering concepts that have not yet been directly validated in scaffold-free cell sheet systems.

Taken together, spatially confined catalysis should be regarded not merely as an enzyme immobilization technique but as a structural engineering strategy for controlling where, when, and to what extent biomechanical reinforcement occurs. Within the proposed Reciprocal Dual-Axis Catalytic Framework, immobilized enzyme-mediated structural catalysis complements nanozyme-mediated redox regulation by directly governing ECM organization, load-bearing architecture, mechanical integration, and long-term structural maturation in diffusion-limited scaffold-free tissues.

### 4.4 Reciprocal coupling between redox regulation and structural catalysis

Although nanozyme-mediated redox regulation and immobilized enzyme-mediated structural catalysis represent distinct catalytic strategies, they should not be interpreted as independent therapeutic interventions. Rather, as illustrated in Figure 2, these catalytic processes operate as functionally coupled regulatory axes that collectively govern scaffold-free cell sheet maturation. Redox regulation primarily preserves biochemical permissiveness by constraining hypoxia-driven oxidative amplification, maintaining cellular viability, and supporting extracellular matrix (ECM) synthesis (Yao et al., 2020; Zhou et al., 2025; Hu S. et al., 2023). Structural catalysis, in contrast, directly reinforces ECM architecture through enzymatic crosslink formation, thereby improving load-bearing capacity and biomechanical integrity. Functional maturation of scaffold-free tissues therefore depends not on either catalytic axis alone but on their reciprocal coordination.

This reciprocal relationship extends beyond simple sequential regulation. Although restoration of redox homeostasis creates favorable conditions for ECM synthesis and crosslink formation, biochemical stabilization alone does not reconstruct mechanical competence once crosslink density and network organization have deteriorated. Conversely, structural reinforcement not only improves tensile strength and resistance to deformation but also modifies mechanotransduction pathways, including YAP/TAZ signaling, thereby influencing cytoskeletal organization, ECM remodeling, and cellular metabolism (Fratzl, 2008; Xiao, 2024; Dupont et al., 2011; Yi et al., 2022). Consequently, structural maturation feeds back into biochemical regulation, while biochemical homeostasis simultaneously supports progressive matrix organization.

This bidirectional interaction becomes particularly important in diffusion-limited scaffold-free tissues, where metabolic stress and mechanical vulnerability coexist (Griffith et al., 2005). Persistent oxidative imbalance disrupts crosslink maturation and matrix organization, whereas mechanical instability further amplifies cellular stress responses and compromises tissue remodeling (Chen J. et al., 2025; Vanderheiden et al., 2015; Cai et al., 2024). Conversely, stabilization of the redox microenvironment promotes more uniform ECM maturation, while mechanically reinforced matrices restore constructive mechanotransduction and facilitate long-term tissue organization (Hu S. et al., 2023; Dupont et al., 2011). Rather than operating through a linear sequence, these processes establish a reciprocal regulatory network in which biochemical stability and biomechanical competence continuously reinforce one another.

Collectively, these observations provide the mechanistic basis for the Reciprocal Dual-Axis Catalytic Framework proposed in this review. As illustrated in Figure 2, biochemical stabilization through nanozyme-mediated redox regulation and biomechanical reinforcement through immobilized enzyme-mediated structural catalysis operate as complementary and mutually dependent regulatory axes. Together, these coupled catalytic processes provide a systems-level strategy for overcoming the biochemical and biomechanical limitations inherent to diffusion-limited scaffold-free cell sheet engineering.

In summary, scaffold-free cell sheet maturation cannot be achieved through intervention along a single catalytic axis. Instead, effective tissue maturation requires coordinated regulation of biochemical homeostasis and biomechanical reinforcement. Nanozyme-mediated redox regulation preserves a permissive biochemical microenvironment that supports sustained cellular viability and extracellular matrix synthesis, whereas immobilized enzyme-mediated structural catalysis establishes the crosslink organization, load-bearing architecture, and mechanical integrity required for long-term tissue stability. Rather than functioning as sequential interventions, these complementary catalytic strategies operate through reciprocal interactions that continuously couple biochemical regulation with structural maturation.

Viewed from this systems-level perspective, the Reciprocal Dual-Axis Catalytic Framework provides more than a conceptual model; it offers a rational engineering strategy for designing scaffold-free tissues capable of overcoming the coupled challenges of diffusion limitation, oxidative stress, and mechanical instability. Importantly, these design principles are not restricted to regenerative medicine. Other dense cellular assemblies generated through scaffold-free biofabrication, including cultured meat and cultured leather, encounter similar constraints associated with diffusion-limited transport, heterogeneous extracellular matrix organization, and progressive mechanical loading. Accordingly, integration of redox regulation with spatially controlled structural catalysis represents a broadly applicable systems engineering framework for guiding the future design of scaffold-free tissues with improved structural maturation, mechanical robustness, and translational potential.

## 5 Future opportunities for reciprocal dual-axis catalytic design beyond regenerative medicine

The Reciprocal Dual-Axis Catalytic Framework proposed in this review was developed to address the coupled biochemical and biomechanical limitations of scaffold-free cell sheet engineering. Nevertheless, the engineering principles underlying this framework are not confined to regenerative medicine. Many emerging scaffold-free biofabrication systems share common challenges associated with diffusion-limited transport, heterogeneous extracellular matrix (ECM) maturation, and progressive mechanical loading. These shared constraints provide opportunities to extend coordinated catalytic regulation to a broader range of high-density tissue fabrication platforms.

Among these emerging applications, cultured meat and cultured leather represent particularly relevant examples because both require the formation of thick, ECM-rich cellular assemblies with uniform structural and mechanical properties (Bettahalli et al., 2011; Lovett et al., 2009; Griffith et al., 2005; McMurtrey, 2016). As construct thickness increases, oxygen and nutrient transport become increasingly diffusion-limited, leading to localized hypoxia, mitochondrial dysfunction, and ROS accumulation (Hamanaka and Chandel, 2009). Simultaneously, heterogeneous ECM deposition and incomplete crosslink formation generate spatial variations in mechanical integrity that become increasingly pronounced as tissue dimensions expand (Shen et al., 2025; Rasouli et al., 2023).

Importantly, successful biofabrication requires more than preservation of cellular viability. In cultured meat, product quality depends on the development of appropriate texture, elasticity, and chewiness, all of which are governed by extracellular matrix organization and crosslink architecture. Likewise, cultured leather requires hierarchical collagen organization, tensile strength, tear resistance, and flexibility that arise from coordinated matrix assembly rather than collagen accumulation alone (Salvatore et al., 2021; Zhai et al., 2022; Li et al., 2022). These mechanical characteristics depend on effective load redistribution, interfacial cohesion, and spatially coordinated matrix maturation.

However, translating this framework beyond regenerative medicine requires careful consideration of the distinct biological and engineering requirements of different scaffold-free biofabrication systems. Compared with therapeutic cell sheets, cultured meat and cultured leather differ substantially in their target cell types, extracellular matrix composition, mechanical performance requirements, and regulatory environments. Despite these application-specific differences, these systems share common engineering challenges associated with diffusion-limited transport, heterogeneous extracellular matrix maturation, and progressive mechanical loading, providing a rationale for extending the proposed conceptual framework.

Within this context, the Reciprocal Dual-Axis Catalytic Framework provides a conceptual engineering strategy for integrating biochemical stabilization with biomechanical reinforcement. Nanozyme-mediated redox regulation may preserve favorable biochemical conditions throughout diffusion-limited constructs, whereas immobilized enzyme-mediated structural catalysis enables localized reinforcement of mechanically vulnerable regions, including sheet interfaces and stress-concentration zones (Shen et al., 2025; McMurtrey, 2016). Although direct experimental validation of this integrated catalytic strategy in cultured meat and cultured leather remains limited, the underlying engineering principles may provide a useful conceptual basis for the future development of high-density scaffold-free biofabrication systems.

Collectively, these considerations suggest that coordinated regulation of redox homeostasis and ECM architecture represents a transferable engineering strategy for overcoming diffusion-induced biochemical instability and mechanical heterogeneity. Extending the Reciprocal Dual-Axis Catalytic Framework beyond regenerative medicine may therefore facilitate the rational design of next-generation scaffold-free biofabrication platforms characterized by improved structural maturation, mechanical robustness, and manufacturing scalability.

## 6 Conclusions and future outlook

Scaffold-free cell sheet engineering has emerged as a promising platform for regenerative medicine and advanced biofabrication, yet its long-term functional maturation remains fundamentally constrained by the coupled effects of diffusion limitation, oxidative stress, extracellular matrix instability, and insufficient mechanical reinforcement. Although these challenges have traditionally been investigated as independent biological processes, increasing evidence suggests that they are functionally interconnected and should be interpreted from a systems-level perspective.

In this review, we propose the Reciprocal Dual-Axis Catalytic Framework as an integrative strategy that combines nanozyme-mediated redox regulation with immobilized enzyme-mediated structural catalysis. Rather than functioning as isolated intervention strategies, these complementary catalytic axes operate through reciprocal interactions that simultaneously regulate biochemical homeostasis and biomechanical maturation. By integrating current knowledge on hypoxia, reactive oxygen species, extracellular matrix remodeling, enzyme-mediated crosslinking, and spatially controlled catalysis, this framework provides a unified conceptual basis for understanding and engineering scaffold-free tissue maturation.

Despite the considerable promise of catalytic regulation, important challenges remain before this framework can be translated into practical tissue engineering strategies. Future studies should prioritize direct validation of coordinated redox and structural catalysis in scaffold-free cell sheet systems, quantitative integration of reaction kinetics, diffusion transport, and tissue mechanics, and development of spatially controlled catalytic platforms capable of sustaining long-term tissue maturation. In parallel, issues related to biocompatibility, manufacturing scalability, and regulatory translation will require systematic investigation before widespread biomedical and industrial implementation can be achieved.

Ultimately, the significance of the proposed framework extends beyond regenerative medicine. As scaffold-free biofabrication continues to evolve toward increasingly complex and high-density cellular systems, coordinated catalytic regulation of biochemical stability and biomechanical competence may provide a broadly applicable systems engineering strategy for overcoming diffusion-limited tissue maturation and enabling the next-generation of engineered biological materials.
